# Is Higher Subjective Fear Predictive of Post-Traumatic Stress Symptoms in a Sample of the Chinese General Public?

**DOI:** 10.3389/fpsyt.2021.560602

**Published:** 2021-05-20

**Authors:** Xin Guo, Tuanjie Liu, Chenqi Xing, Yan Wang, Zhilei Shang, Luna Sun, Yanpu Jia, Lili Wu, Xiong Ni, Weizhi Liu

**Affiliations:** ^1^Lab for Post-traumatic Stress Disorder, Faculty of Psychology and Mental Health, Naval Medical University, Shanghai, China; ^2^The Battalion 3 of Cadet Brigade, School of Basic Medicine, Naval Medical University, Shanghai, China; ^3^Department of Neurology, Wusong Central Hospital, Shanghai, China; ^4^The Emotion & Cognition Lab, Faculty of Psychology and Mental Health, Naval Medical University, Shanghai, China; ^5^Department of Hematology, Changhai Hospital, Naval Medical University, Shanghai, China

**Keywords:** subjective fear, post-traumatic stress symptoms, COVID-19, sleep quality, general public health

## Abstract

**Background:** COVID-19 has taken a huge toll on medical resources and the economy and will inevitably have an impact on public mental health. Post-traumatic stress disorder (PTSD), as the most common mental illness after an epidemic, must be seriously addressed. This study aimed to investigate the subjective fear of the Chinese general public during COVID-19 and to explore how it affected the development of PTSD.

**Methods:** An online questionnaire survey was conducted among 1,009 people from January 30 to February 14, 2020 (about 1 month after the COVID-19 outbreak). The subjective fear was measured by a self-reported single-choice question. Four items from the Pittsburgh Sleep Quality Index (PSQI) were selected to measure the subjects' sleep quality. Their post-traumatic stress symptoms (PTSS) were measured by the PTSD Checklist for DSM-5 (PCL-5). Pearson correlation, hierarchical multivariate regression analysis, multiple mediator model, and bootstrapping were used in statistical analyses.

**Results:** Different people showed different levels of subjective fear in response to the outbreak. There was a significant positive correlation between subjective fear and the total score of PCL-5 (*R* = 0.513, *P* < 0.01), meaning that the higher the degree of subjective fear, the more severe the symptoms of post-traumatic stress are. Subjective fear was an important predictor of PTSS, accounting for 24.3% of the variance. The total effect of subjective fear on PCL-5 scores was significant (total effect = 7.426, *SE* = 0.405, 95% CI = 6.631–8.221). The total indirect effect of subjective fear on PCL-5 scores through sleep quality was also significant (total indirect effect = 1.945, *SE* = 0.258, 95% CI = 1.436–2.470).

**Conclusions:** Subjective fear has an important predictive effect on PTSS. In addition to the direct effect, our findings firstly demonstrate the mediating role of sleep quality in the relationship between subjective fear and PTSS.

## Introduction

The novel coronavirus pneumonia called COVID-19, which is a disease caused by SARS-CoV-2, has been spreading worldwide for nearly a year. The World Health Organization (WHO) first learned of this new virus on December 31, 2019, following a report of a cluster of cases of “viral pneumonia” (1). As of January 19, 2021, the WHO reported that the cumulative numbers had grown to over 93 million reported cases and over two million deaths globally since the start of the pandemic (2). People around the world are working to fight off the epidemic, and COVID-19 vaccines are being inoculated. However, vaccines can be a useful technique to complement but not replace preventive health protocols, which have been shown to suppress transmission and save lives. Humanity still has a long way to go to fight off this disease (3). The clinical manifestations of the disease usually start after <1 week, consisting of fever, cough, nasal congestion, fatigue, and other signs of upper respiratory tract infections (4). Such outbreaks not only influence the economic development of society but also inevitably lead to public mental health problems (5). During the COVID-19 outbreak, researchers have shown an increased interest in mental health problems of health care workers and survivors; however, very few studies have focused on the general public in China (6–9).

Psychological distress, anxiety, depression, and post-traumatic stress disorder (PTSD) have been generally recorded among populations exposed to various infectious diseases (10–21). Ko et al. reported the psychosocial impact among the public of the Severe Acute Respiratory Syndrome (SARS) in Taiwan (22). Mak et al. indicated that one-fourth of the patients among SARS survivors had post-traumatic stress symptoms (PTSS) (23). Jalloh et al. assessed symptoms of anxiety, depression, and PTSD in the general population of Sierra Leone after 1 year of the Ebola outbreak and found that the prevalence of PTSS was 76% (24). PTSS was the most common of these possible mental disorders according to previous studies (25), which required researchers to devote more time and energy similarly at the outbreak of COVID-19.

There are many influencing factors of PTSS, such as the severity of the epidemic, gender, and personality (26). Among them, emotion-related factors, especially fear, which is a common response in PTSS, have raised a concern for many researchers (27). There were plenty of studies showing that subjective fear in the context of an outbreak had a significant impact on the development of PTSD (28). The processing and regulation of fear were key components of PTSD (29). Failure to intervene well with people's subjective fear may increase their risk of developing PTSD, which in turn affects social productivity. However, how subjective fear affects the development of PTSD remains unresolved.

This study aimed to investigate the subjective fear of the Chinese general public during COVID-19 and to explore how it affects the development of PTSD. The findings of the current study provide important reflections on the implications of similar infectious diseases in the future.

## Methods

### Participants

A cross-sectional design was adopted in the study. From January 30 to February 14, 2020 (about 1 month after the COVID-19 outbreak), an online questionnaire survey was conducted in the mainland of China. The survey was anonymous, and participants completed the questionnaire independently according to their psychological status in the past 1–2 weeks.

**Figure 1:**
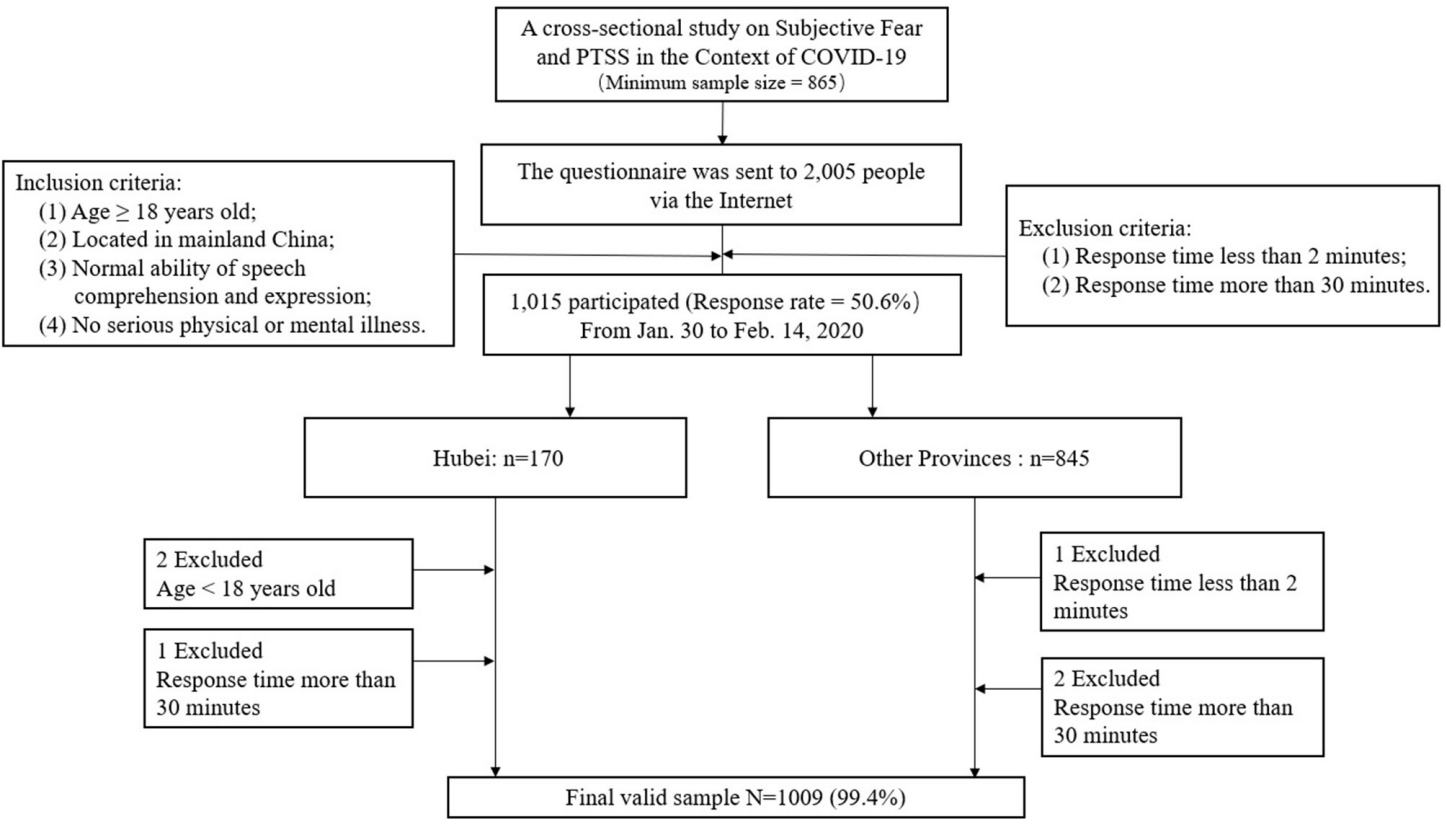
Flowchart depicting the passage of participants.

The inclusion criteria were (1) being ≥18 years of age; (2) being located in mainland China; (3) having normal ability in language, comprehension, and expression; and (4) having no serious physical or mental disorder (like schizophrenia). The exclusion criteria were (1) response time <2 min and (2) response time more than 30 min. Too long or too short a response indicated that they were more likely to take the survey less seriously. The questionnaire was sent to 2,005 people who agreed to participate in the study via the Internet. A total of 1,015 people participated in the survey, with a response rate of 50.6%, including 170 from Hubei province (two were under 18 years old, and one's response time was more than 30 min) and 845 from other provinces (one who responded for <2 min, and two who responded for more than 30 min). As a result, 1,009 valid questionnaires were obtained, with a response rate of 99.4%. The flowchart of participants is shown in Figure 1.

### Measures

The basic demographic information was first collected, including age, gender, education level, occupation, location, exposure history of Wuhan, and classification of the susceptible population (including vulnerable population and the general population; close contact with a patient; close contact with the patient, medical care, and related personnel; patients with suspected infection and patients with confirmed infection).

The subjective fear was measured by a self-reported single-choice question, “How would you rate the level of fear you feel at the moment,” and rated from “not at all” to “extremely.”

Four items from the Pittsburgh Sleep Quality Index (PSQI) were selected to measure the subject's sleep quality, including the following: (1) How would you rate the degree of your sleep quality? (2) Do you have trouble falling asleep within 30 min? (3) Do you wake up easily at night or too early in the morning? (3) How long do you usually sleep each night these days?

The PTSD Checklist for DSM-5 (PCL-5) was used to measure PTSS. It takes ~5–10 min to complete (30). Based on a 5-point Likert-type scale from 0 (not at all) to 4 (extremely), participants were asked to indicate how much discomfort they had experienced due to a particular symptom in the past week (including the current day). A total symptom severity score (range 0–80) can be obtained by summing the scores for each of the 20 items. DSM-5 symptom criterion severity scores can be obtained by summing the scores for the items within a given cluster, i.e., criterion B (items 1–5), criterion C (items 6 and 7), criterion D (items 8–14), and criterion E (items 15–20). A cutoff point of more than 33 points was considered to confirm a provisional diagnosis (31). A higher score indicated more severe PTSS.

Additionally, the investigation came at a time when the COVID-19 outbreak was spreading across China, putting the Chinese general public at high risk of contracting this deadly disease. According to the Life Events Checklist for DSM-5 (LEC-5) of PCL-5, this experience was a traumatic event. The instruction in our survey was delivered: this is a questionnaire to assess the extent to which the COVID-19 outbreak has affected you.

### Statistical Analysis

**Figure 2:**
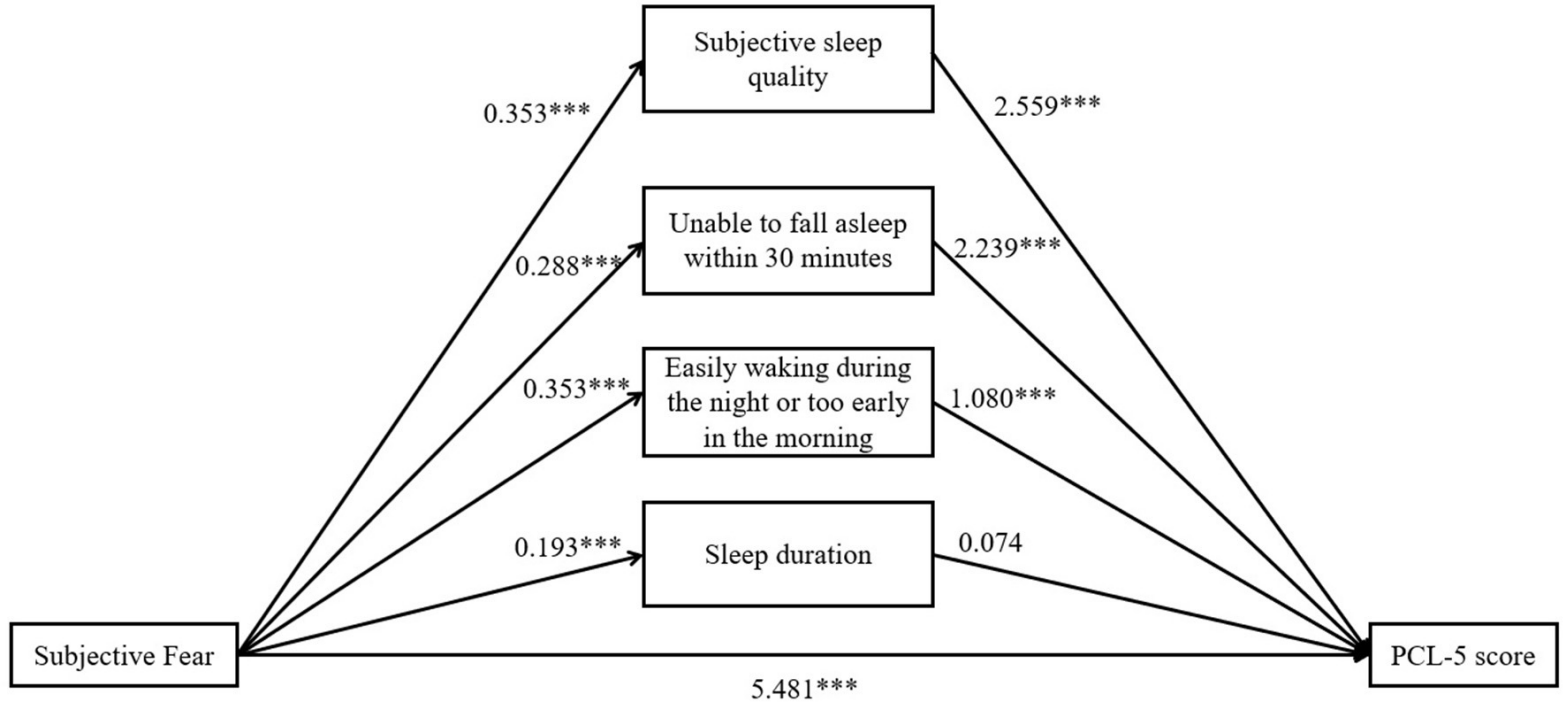
Multiple mediator model. All the coefficients were unstandardized beta. ***P < 0.001.

Statistical analyses were conducted by using SPSS (Armonk, NY, USA), version 20, with an SPSS macro named PROCESS 3.3. Descriptive statistical analysis was conducted to illustrate the demographic characteristics. Independent-samples *t*-test and analysis of variance were used to detect the difference on subjective fear among different subgroups. Pearson correlation was used to identify factors associated with subjective fear. A value of *P* < 0.05 was considered statistically significant. In order to investigate whether subjective fear has an impact as an independent predictor or a moderator on PTSS, a hierarchical multivariate regression analysis (see **Table 4**) was performed. According to the results of the regression analysis, a multiple mediator model was conducted to examine the relative strength of the mediation effect of sleep quality on subjective fear and PTSS (see **Table 4** and Figure 2). In this model, subjective fear was the predictor variable, sleep quality was a mediator, and the PCL-5 score was the outcome variable. Bootstrapping (with 5,000 bootstrap samples) was used to estimate 95% confidence intervals (CIs) and confirm the indirect (mediating) effect. Age, gender, education level, occupation, location, exposure history of Wuhan, and classification of the susceptible population were controlled in the analyses.

## Results

### Demographics Characteristics

Demographic information and analysis of variance of subjective fear are shown in Table 1. The average age of subjects was 38.3 ± 11.5. Nearly a third of the participants (31.0%) were 41–50 years old, more than a quarter (27.5%) were 31–40 years old, and nearly a quarter (24.6%) were 21–30 years old. The majority of participants (64.4%) were female, 64.3% were University or college graduates, 16.6% lived in Hubei province, and 17.3% had an exposure history of Wuhan, which was the worst affected area by the epidemic at that time. Among the participants, the majority (83.4%) were susceptible groups or the Chinese general public, classified as low-risk public; 2.8% were close contacts or suspected patients, classified as high-risk public; and 13.8% were health care workers.

**Table 1:** Demographic information and analysis of variance of subjective fear.

	***N***	**%**	**Subjective Fear Scores**		***F*/*t***	***P*-value**
			**MEAN**	***SD***		
**Total**	1,009	100.0	2.02	0.73		
**Age**						
≤ 20	39	3.9	1.77	0.43	2.654	0.022
21–30	248	24.6	2.03	0.67		
31–40	277	27.5	2.13	0.86		
41–50	313	31.0	1.98	0.67		
51–60	105	10.4	1.95	0.74		
>60	27	2.7	1.96	0.76		
**Gender**						
Male	359	35.6	1.87	0.60	−5.034	<0.001
Female	650	64.4	2.11	0.79		
**Education Level**						
High school or below	136	13.5	1.82	0.63	6.596	0.001
University or college	649	64.3	2.07	0.75		
Post-graduate or above	224	22.2	2.03	0.72		
**Locations**						
Hubei	167	16.6	2.11	0.76	1.735	0.083
Other provinces	842	83.4	2.01	0.73		
**Exposure history of Wuhan**						
No	834	82.7	2.00	0.72	−1.672	0.096
Yes	175	17.3	2.11	0.80		
**Classification of susceptible population**						
Health care workers	139	13.8	2.07	0.70	1.818	0.163
Low-risk public	842	83.4	2.01	0.73		
High-risk public	28	2.8	2.25	1.08		

The mean score on subjective fear was 2.02 [standard deviation (*SD*) = 0.73]. Results of the variance showed that there were significant differences between subjective fear and demographic characteristics, including age (*P* = 0.022), gender (*P* < 0.001), and education level (*P* = 0.001). Participants who were aged 31–40 years, were female, were University or college-educated, were living in Hubei province, had an exposure history of Wuhan, and were classified as high-risk public had a higher level of subjective fear.

### Prevalence of PTSS

The score of PCL-5 and prevalence of PTSS are shown in Table 2. The mean score on PCL-5 was 12.94 (*SD* = 10.81). The cutoff score is 33. According to this scale, 57 out of 1,009 had a positive score, which indicated the prevalence of PTSS as 5.6%. The avoidance symptoms assessed by criterion C were less common 1 month after the epidemic [with a mean score of 1.15 (*SD* = 1.65)] compared with other PTSS. The average scores were 3.84 (*SD* = 3.48) for criterion B, 3.99 (*SD* = 4.09) for criterion D, and 3.96 (*SD* = 3.67) for criterion E. Forty-seven percent of the participants reported at least one criterion B (intrusion) symptom, and the prevalence rates of symptom in criterion C (avoidance), criterion D (negative changes in thoughts and mood), and criterion E (hyperarousal) were 19.4, 44.8, and 40.7%, respectively.

**Table 2:** Scores of PCL-5 and prevalence of PTSS.

	**MEAN/*N***	***SD*/%**
**PCL-5 scores**		
Total scores	12.94	10.81
Criterion B (intrusion)	3.84	3.48
Criterion C (avoidance)	1.15	1.65
Criterion D (negative changes in thoughts and mood)	3.99	4.09
Criterion E (hyperarousal)	3.96	3.67
**Prevalence of symptoms**		
PTSS	57	5.6
Criterion B (intrusion)	474	47.0
Criterion C (avoidance)	196	19.4
Criterion D (negative changes in thoughts and mood)	452	44.8
Criterion E (hyperarousal)	411	40.7

*PTSS was determined by PCL-5 scores by a cutoff of 33. Criteria B, C, D, and E symptoms indicated that there was at least one item of the items in the criterion that participants rated 2 or higher*.

### Correlation of Subjective Fear, Sleep Quality, and PTSD

The correlation between subjective fear, sleep quality, and PTSD is presented in Table 3. There was a significant positive correlation between subjective fear and the total score of PCL-5 (*R* = 0.513), as well as the four symptom clusters (intrusion, avoidance, negative changes in thoughts and mood, and hyperarousal) of PCL-5, meaning that a higher level of subjective fear was associated with the severity of PTSS. The correlation between subjective fear and sleep quality was also significant. In addition, the PCL-5 scores were positively correlated with subjective sleep quality, inability to fall asleep within 30 min, easy waking during the night or too early in the morning, and sleep duration.

**Table 3:** Pearson correlation coefficient of subjective fear, sleep quality, and PTSD.

	**1**	**2**	**3**	**4**	**5**	**6**	**7**	**8**	**9**	**10**
1. Subjective fear	1									
2. PCL-5 scores	0.513^**^									
3. PCL-5 (intrusion)	0.576^**^	0.828^**^								
4. PCL-5 (avoidance)	0.312^**^	0.668^**^	0.577^**^							
5. PCL-5 (negative changes in thoughts and mood)	0.377^**^	0.878^**^	0.557^**^	0.469^**^						
6. PCL-5 (hyperarousal)	0.405^**^	0.882^**^	0.610^**^	0.449^**^	0.733^**^					
7. Subjective sleep quality	0.348^**^	0.499^**^	0.453^**^	0.273^**^	0.385^**^	0.489^**^				
8. Inability to fall asleep within 30 min	0.236^**^	0.469^**^	0.379^**^	0.233^**^	0.357^**^	0.521^**^	0.646^**^			
9. Easy waking during the night or too early in the morning	0.250^**^	0.400^**^	0.350^**^	0.160^**^	0.311^**^	0.427^**^	0.512^**^	0.515^**^		
10. Sleep duration	0.170^**^	0.241^**^	0.212^**^	0.077^*^	0.190^**^	0.264^**^	0.429^**^	0.325^**^	0.391^**^	1

* *P < 0.05;*

** *P < 0.01*.

### Mediating Role of Sleep Quality in the Relationship of Subjective Fear and PTSS

The results of hierarchical multiple regression analysis are presented in Table 4. In the first step, age, gender, and education level were added into the model, and the education level was transformed into two dummy variables (high school or below vs. University or college and post-graduate or above vs. University or college), with University or college as the reference group. In the second step, locations, exposure history of Wuhan, and classification of susceptible population were added as regressors, and classification of susceptible population was transformed into two dummy variables (high-risk public vs. health care workers and low-risk public vs. health care workers). In the third step, subjective fear entered the model. Finally, we put four dimensions of sleep quality into the model to see if sleep quality affected the relationship between subjective fear and PTSS. Model 1 accounted for 1.4% of the variance in PTSS, in which the main effects of female gender and education level of high school or below were significant in predicting the severity of PTSS. In model 2, the main effects of being currently in Hubei and high-risk public were significant, which accounted for the additional 1.9% of variance in PTSS. Model 3 accounted for the additional 24.3% of variance; subjective fear was an important predictor of PTSS. In model 4, the main effects of subjective sleep quality, inability to fall asleep within 30 min, and easy waking during the night or too early in the morning were significant, while sleep duration was not significant, which accounted for the additional 15.7% of the variance in PTSS. Meanwhile, when the four dimensions of sleep quality entered the model, the unstandardized regression coefficient of subjective fear dropped from 7.426 to 5.481. The possibility that sleep quality influences the relationship between subjective fear and PTSS may be the main reason for this result.

**Table 4:** Regression analyses with PCL-5 score as the dependent variable (*n* = 1,009).

**Variables**	**PCL-5 score**			***R*^**2**^**	**Adjusted *R*^**2**^**	***R*^**2**^ change**	***F***	***P*-value**
	***B***	**β**	***t***					
**Step 1**								
Age	−0.006	−0.007	−0.207	0.014	0.010	0.014	3.521	0.007
Female vs. Male	2.172	0.096	3.057^**^					
High school or below vs. University or college	−2.036	−0.064	−1.982^*^					
Post-graduate or above vs. University or college	−0.626	−0.024	−0.750					
**Step 2**								
Age	−0.013	−0.014	−0.443	0.033	0.025	0.019	4.223	<0.001
Female vs. Male	2.351	0.104	3.324^**^					
High school or below vs. University or college	−2.235	−0.071	−2.156^*^					
Post-graduate or above vs. University or college	−0.605	−0.023	−0.725					
Currently in Hubei vs. Other Provinces	3.284	0.113	2.601^**^					
Exposure history of Wuhan Yes vs. No	−0.434	−0.015	−0.342					
High-risk public vs. Health care workers	4.715	0.072	2.037^*^					
Low-risk public vs. Health care workers	1.804	0.062	1.802					
**Step 3**								
Age	−0.023	−0.024	−0.881	0.276	0.270	0.243	42.341	<0.001
Female vs. Male	0.523	0.023	0.843					
High school or below vs. University or college	−0.434	−0.014	−0.481					
Post-graduate or above vs. University or college	−0.470	−0.018	−0.651					
Currently in Hubei vs. Other Provinces	2.640	0.091	2.415^*^					
Exposure history of Wuhan Yes vs. No	−0.690	−0.024	−0.629					
High-risk public vs. Health care workers	3.684	0.056	1.838					
Low-risk public vs. Health care workers	1.991	0.068	2.298^*^					
Subjective fear	7.426	0.504	18.329^**^					
**Step 4**								
Age	−0.022	−0.023	−0.896	0.433	0.426	0.157	58.508	<0.001
Female vs. Male	−0.082	−0.004	−0.149					
High school or below vs. University or college	−0.101	−0.003	−0.125					
Post-graduate or above vs. University or college	−0.155	−0.006	−0.241					
Currently in Hubei vs. Other Provinces	1.980	0.068	2.040^*^					
Exposure history of Wuhan Yes vs. No	−0.715	−0.025	−0.733					
High-risk public vs. Health care workers	3.616	0.055	2.033					
Low-risk public vs. Health care workers	2.521	0.087	3.241^**^					
Subjective fear	5.481	0.372	14.353^***^					
Subjective sleep quality	2.559	0.185	5.350^***^					
Inability to fall asleep within 30 min	2.239	0.199	5.987^***^					
Easy waking during the night or too early in the morning	1.080	0.109	3.640^***^					
Sleep duration	0.074	0.006	0.209					

*B, unstandardized beta; β, standardized regression weight. The education level was transformed into two dummy variables (high school or below vs. University or college and post-graduate or above vs. University or college) with the University or college as the reference group. Classification of susceptible population was transformed into two dummy variables (high-risk public vs. health care workers and low-risk public vs. health care workers)*.

* *P < 0.05;*

** *P < 0.01; and*

*** *P < 0.001*.

Hence, multiple mediator analyses were then conducted to test the hypothesis that sleep quality mediated the relationship between subjective fear and PTSS. The parameters estimated for the total, direct, and specific indirect effects with bias-corrected 95% CI for the multiple mediator model are reported in Table 5. Subjective sleep quality, inability to fall asleep within 30 min, easy waking during the night or too early in the morning, and sleep duration were added simultaneously into the model, with age, gender, education level, location, exposure history of Wuhan, and classification of susceptible population as covariates; see Figure 2. The total effect of subjective fear on PCL-5 scores was significant (total effect = 7.426, *SE* = 0.405, 95% CI = 6.631–8.221). The total indirect effect of subjective fear on PCL-5 scores through sleep quality was also significant (total indirect effect = 1.945, *SE* = 0.258, 95% CI = 1.436–2.470). Subjective sleep quality (indirect effect = 0.904, *SE* = 0.186, 95% CI = 0.557–1.270), inability to fall asleep within 30 min (indirect effect = 0.645, *SE* = 0.159, 95% CI = 0.354–0.987), and easy waking during the night or too early in the morning (indirect effect = 0.382, *SE* = 0.127, 95% CI = 0.164–0.652) were the significant mediators between subjective fear and PTSS. However, sleep duration did not significantly mediate the relationship between subjective fear and PTSS (*P* > 0.05). The model significantly explained 43.3% (*R*^2^, *P* < 0.001) of the variance in PCL-5 scores.

**Table 5:** Unstandardized total, direct, and indirect effect of subjective fear on PCL-5 score through sleep quality.

**Effects on PCL-5 score**	**Variables**	**Effects**	**SE**	***t***	***P*-value**	**Bootstrap 95% CI**	
						**Lower**	**Upper**
Total effect (c)	Subjective fear	7.426	0.405	18.330	<0.001	6.631	8.221
Direct effect (c′)	Subjective fear	5.481	0.382	14.353	<0.001	4.732	6.230
Indirect effect	Subjective sleep quality	0.904	0.186			0.557	1.270
	Inability to fall asleep within 30 min	0.645	0.159			0.354	0.987
	Easy waking during the night or too early in the morning	0.382	0.127			0.164	0.652
	Sleep duration	0.014	0.071			−0.127	0.154
	Total indirect effect	1.945	0.258			1.436	2.470

## Discussion

As the whole world is under the shade of COVID-19, the fear caused by it spreads rapidly through a multitude of channels. Data from recent public opinion polls showed that COVID-19 was generating subjective fear (32). In the United States and Canada, the majority of people are concerned about the sustained transmission of the disease and the ability of the government to respond to the growing number of new cases (33). The subjective fear of COVID-19 arose from the underlying anxiety with unknown reason, and it will be further fueled when infection control techniques and restrictive practices such as quarantine and isolation are employed to protect the public's health (33). Emphasizing the importance of subjective fear was necessary. However, there has been no study exploring PTSS from the aspect of subjective fear until now. Therefore, the current study aimed to investigate the important effects of subjective fear on PTSS during the COVID-19 outbreak.

As of February 20, 55,924 laboratory-diagnosed cases have been reported, of which the majority (77.8%) were between 30 and 69 years old and 51.1% were male, according to the China–WHO Joint Investigation of Corona Virus Disease (COVID-19) (34). Generally speaking, people between the ages of 21 and 40 years are breadwinners of their families, so the impact on the whole family is devastating once they are infected. Therefore, it was not hard to understand the result that people within this age had higher levels of subjective fear. According to the data, people aged 31–40 years scored highest in subjective fear, which may lead them to take more aggressive treatment, and this may explain why the average age of participants was relatively low in the current study. Although there was no significant difference in susceptibility to the COVID-19 between males and females, females showed greater subjective fear in response to the outbreak, which may be related to gender socialization and self-efficacy, consistent with previous researches (35). Furthermore, people with higher levels of education had higher levels of subjective fear, which was also in line with previous researches on other aspects of fear (36). This may be related to access to information and the awareness of disease severity. Higher-educated people tended to have more access to information, making them more likely to be exposed to rumors. This reminds us that the media should pay more attention to scientificity and authenticity when reporting related information. Given that Hubei accounted for 77% of the country's cases (34), it was not surprising that those at high risk, such as those living in Hubei or having a history of exposure to Wuhan, had a higher level of subjective fear. On the contrary, health care workers showed lower subjective fear, which may be related to their more professional self-protection knowledge and skills.

A survey conducted during the SARS quarantine in Toronto, Canada, showed that symptoms of PTSD were observed in 28.9% of respondents (37). Results showed that 27% of clinical concerns tested positive for PTSD and 16% met levels of probable PTSD (24). The prevalence of PTSS in the present study was found to be 5.6%, which was lower than that of previously reported levels. This may be because PTSS do not fully manifest 1 month after the outbreak. However, a survey on the psychological status of home-quarantined Chinese University students during COVID-19 showed that the prevalence of PTSD was 2.7% (38), which was lower than our results. The difference may be explained in part by the education level or age of students. Further studies which need to be undertaken should take these variables into account. In addition, only 19.4% of participants reported at least one criterion C (avoidance) symptom, which was lower compared with other criteria, suggesting that when people are faced with COVID-19, they did not have significant avoidance but tended to have more intrusion, hyperarousal, and negative changes in thoughts and mood.

There was a close relationship among subjective fear, sleep quality, and PTSS, which was in line with the results of previous studies (39–42). For instance, Zuj et al. have found that greater sleep disturbance and longer sleep onset latency facilitated SCR-specific fear reinstatement in PTSS (39). In addition, Fan et al. found that sleep disturbance predicted the development and persistence of PTSS and depressive symptoms (40), suggesting that sleep quality played an important role in both the treatment and prevention of PTSD. Previous studies of other trauma exposures indicated that fear was an important predictor of PTSD symptoms. For example, the research of Salcioglu et al. suggested that anticipatory fear predicted PTSS in domestic violence survivors (41). Moreover, the research of Çapik et al. found that fear of childbirth was a predictor of PTSD after childbirth (42). However, the way in which subjective fear affected PTSD symptoms was not clear; further analysis on this topic was therefore suggested.

Considering the analysis of hierarchical multiple regression, model 1 suggested that the main effects of female gender and education level of high school or below were significant in predicting the severity of PTSD symptoms, which was consistent with the previous studies. The prevalence rate of PTSD is 5–10%, which is twice as high for women as for men (43). It could have resulted from the greater exposure associated with the susceptible population of PTSD, and it might also be associated with sex-specific risk factors (44). Currently, there was no sufficient evidence showing that higher education led to more PTSS, and we speculated that it may be also related to subjective fear. But more researches are needed. With Hubei being the epi-affected area at that time, it is understandable that local residents may have had more severe traumatic experiences, leading to more severe PTSS. Many previous studies also indicated that the severity of trauma was an important predictor of PTSS (45, 46). In model 2, the main effects of being currently in Hubei and a high-risk public were significant. The results were in accord with the status quo of the corresponding subjective fear, suggesting the important influence of subjective fear on PTSD symptoms (47–50).

When subjective fear was added into the model, it showed that subjective fear was an important predictor of PTSS, which was in line with our expectations that subjective fear plays an important role in the development of PTSS. The existing studies, showing subjective fear in the context of epidemics, had a significant impact on the development of PTSS, providing evidence to support our results. In addition, both present results and previous studies indicated that females showed greater subjective fear in response to the outbreak. This possibly further supported the result of females being more likely to develop PTSS and explained why gender was not a significant factor in model 3. Therefore, paying more attention to the current emotional mental response during the COVID-19 outbreak is important for the Chinese general public to cope with PTSS (51, 52). Meanwhile, given that the relationship between sleep and PTSS may be bidirectional, the relationship between subjective fear, gender, and PTSS remains to be further elucidated.

It is worth noting that, in addition to the direct effect, the current study also firstly illustrated the mediating role of sleep quality in the relationship between subjective fear and PTSS. To be more specific, the sleep-related indirect effects were significant according to Figure 2, which were subjective sleep quality, inability to fall asleep within 30 min, and easy waking during the night or too early in the morning. Therefore, sleep quality as the mediator between subjective fear and PTSS could be a possible factor, if not the only one (53), causing PTSD during the outbreak of COVID-19. More targeted interventions can be implemented based on this aspect.

Despite several important results of this study, there are some limitations that warrant discussion. First, the cross-sectional design makes it difficult to identify causal relationships among all factors. Therefore, a longitudinal study is recommended. In addition, our survey was conducted through online questionnaires, so our sample was not necessarily nationally representative. The sample had a higher proportion of respondents with lower mean age and higher education level compared with the general population, which can lead to a degree of bias in the results. Among other factors, the lack of informatics skills among senior/less educated people may be preventing them from filling in the survey, lowering the representativeness of the sample. Fortunately, education had little effect on PTSS compared to subjective fear, suggesting that this may reduce this potential recall bias. Besides, this study is devoted to exploring the predictive effect of sleep on PTSS, and previous studies also mentioned that sleep quality may be a consequence/symptom of post-traumatic stress. The relationship between sleep and PTSS may be bidirectional, so more studies are needed.

## Conclusion

In conclusion, our findings first demonstrate the important predictive effects of subjective fear in PTSS and the mediating role of sleep quality in the relationship between subjective fear and PTSS. The regulation of fear and sleep is crucial for the prevention and intervention of PTSS during the outbreak of COVID-19, providing important implications for the future emergence of similar infectious diseases.

## Data Availability Statement

The raw data supporting the conclusions of this article will be made available by the authors, without undue reservation.

## Ethics Statement

The studies involving human participants were reviewed and approved by Naval Medical University. Written informed consent for participation was not required for this study in accordance with the national legislation and the institutional requirements.

## Author Contributions

XG, TL, and CX contributed to the writing and statistical analysis of this work. WL and XN led the study, including putting forward, and carrying out the study. YW, ZS, LS, YJ, and LW contributed to the investigation and collection of all data. All authors are accountable for all aspects of the work and in ensuring that questions related to the accuracy or integrity of any part of the work are appropriately investigated and resolved.

## Conflict of Interest

The authors declare that the research was conducted in the absence of any commercial or financial relationships that could be construed as a potential conflict of interest.
